# Application of a new real-time polymerase chain reaction assay for surveillance studies of lymphocystis disease virus in farmed gilthead seabream

**DOI:** 10.1186/s12917-016-0696-6

**Published:** 2016-04-06

**Authors:** Estefania J. Valverde, Irene Cano, Alejandro Labella, Juan J. Borrego, Dolores Castro

**Affiliations:** Departamento de Microbiología, Universidad de Málaga, 29071 Málaga, Spain; CEFAS Weymouth Laboratory, Weymouth, Dorset DT4 8UB UK

**Keywords:** Lymphocystis disease virus, Virus surveillance, Farmed gilthead seabream, Real-time PCR

## Abstract

**Background:**

Lymphocystis disease (LCD) is the main viral infection reported to affect cultured gilthead seabream (*Sparus aurata*) in Europe. The existence of subclinical Lymphocystis disease virus (LCDV) infection in this fish species has been recognised by using polymerase chain reaction (PCR)-based methods. Nevertheless, these methods do not provide quantitative results that can be useful in epidemiological and pathological studies. Moreover, carrier fish have been involved in viral transmission, therefore the use of specific and sensitive diagnostic methods to detect LCDV will be relevant for LCD prevention.

**Results:**

We have developed a real-time PCR (qPCR) assay to detect and quantify LCDV. The assay was evaluated for viral diagnosis in surveillance studies in gilthead seabream farms, and also to identify viral reservoirs in a hatchery. The prevalence of LCDV infection in the asymptomatic gilthead seabream populations tested varied from 30 to 100 %, including data from one farm without previous records of LCD. Estimated viral load in caudal fin of subclinically infected fish was two to five orders of magnitude lower than in diseased fish. The qPCR assay allowed the detection of carrier fish in broodstock from a farm with a history of clinical LCD in juvenile fish. In addition, the quantitative detection of LCDV was achieved in all samples collected in the hatchery, including fertilized eggs, larvae and fingerlings, and also rotifer cultures and artemia metanauplii and cysts used for larval rearing.

**Conclusions:**

The qPCR assay developed in this study has proved to be a rapid, sensitive, and reliable method for LCDV diagnosis, which could be valuable to identify LCDV reservoirs or to study viral replication in gilthead seabream.

## Background

Lymphocystis disease (LCD) affects a wide variety of wild and cultured fish species with an extensive geographical distribution [1]. In Southern Atlantic and Mediterranean aquaculture, this disease is the main viral infection reported to affect cultured gilthead seabream (*Sparus aurata*) [2–4]. The typical sign of lymphocystis disease is the presence of small pearl-like nodules on the skin and fins of affected fish, that may occur singly or more generally grouped in raspberry-like clusters of tumorous appearance [5, 6]. Although this disease is rarely fatal, fish showing these symptoms appear unsightly and cannot be commercialized, causing important economic losses [7]. Moreover, in fish farms, LCD outbreaks may favour secondary bacterial infections, cannibalism and/or parasitic infestations, factors that may increase mortality rates [4, 8–10].

The etiological agent of LCD is the Lymphocystis disease virus (LCDV), a member of the genus *Lymphocystivirus*, family *Iridoviridae*. On the basis of the major capsid protein (MCP) gene sequence, nine genotypes of *Lymphocystivirus* have been proposed [11–15]. All LCDV isolates from gilthead seabream and Senegalese sole (*Solea senegalensis*) sequenced so far are included in genotype VII [10, 14, 16].

The only adequate measure for LCD prevention in the aquaculture systems is the use of general prophylactic practices, such as good husbandry practices, reduced stocking density and the virological control of fish to be introduced in the farm facilities in order to detect carrier fish [1]. These animals may pose a risk for the introduction of LCDV in fish farms, as direct contact between fish specimens is considered the main route of LCDV spreading [5]. More recently, asymptomatic carrier breeders have also been involved in LCDV transmission to fish larvae [17]. The detection of subclinical viral infections in carrier fish requires the use of sensitive diagnostic methods [18]. In this context, polymerase chain reaction (PCR)-based methods have proved to be adequate for LCDV detection in apparently healthy fish [10, 16, 19–21]. The PCR-hybridization assay developed by Cano et al. [19] not only allowed the detection of LCDV in carrier gilthead seabream, but also in rotifer and artemia used as live food for larval stages, which makes it a valuable tool for the detection of other potential LCDV foci in fish farms [17, 22]. Nevertheless, this assay is relatively time-consuming, not readily applied to screening large sample numbers, and does not provide quantitative results, that can be useful in epidemiological and pathological studies of LCDV.

Real-time PCR (qPCR) has been used for the detection and quantification of numerous viral fish pathogens, including LCDV in yellow perch (*Perca flavescens*) [15], and has been proved to be useful to overcome the disadvantages of conventional PCR above mentioned [23–25]. Recently, a qPCR assay has been developed and applied for the detection and quantification of LCDV in a low number of samples of diseased and recovered gilthead seabream. Nevertheless, the use of the assay for LCDV monitoring in different stages of the fish production cycle was not investigated [26].

The objective of this study was to establish the applicability of a new qPCR assay for LCDV diagnosis in surveillance studies. In addition, this assay has been evaluated using samples from a gilthead seabream hatchery, in order to demonstrate its utility to detect several sources of LCDV in the fish farm.

## Methods

### Ethics statement

Fish farms owners accepted to participate in the study, and specialized personnel in each farm facility carried out the sampling procedures described below. Fish used in this study have been treated in compliance with the Spanish legislation (Law 32/2007, and RD 53/2013).

### Sample collection and DNA extraction

Juvenile gilthead seabream specimens were sampled at four aquaculture farms. In two of these farms (farms A and B), a total of 11 diseased and 25 asymptomatic (i.e., without signs of lymphocystis disease) fish were collected during an outbreak of LCD, whereas in the other two (farms C and D), only asymptomatic fish were observed, and 24 of them were collected. Juvenile fish were euthanized by anaesthetic overdose (MS-222) (Sigma-Aldrich, St. Louis, MO, USA) before sampling. Samples of caudal fin (approximately 1 cm^2^ in size) were aseptically cut off, frozen immediately at -20 °C in dry ice and sent to the lab. In addition, 9 gilthead seabream samples were collected at the hatchery in another farm suffering LCD outbreaks over several years (farm E). Samples consisted of fertilized eggs (one sample of 100 mg), larvae (4 pools of 10–15 animals), and fingerlings (up to 2 g of weight) (4 pools of 5–10 animals). No LCD clinical signs were observed in any of the sampled fingerlings. Larvae and fingerlings were also euthanized by anaesthetic overdose. One sample of each rotifers (100 mg), commercial artemia cysts (100 mg), decapsulated artemia cysts (100 mg) and artemia metanauplii (100 mg) used as live food for larvae were also collected. These samples were washed with sterile seawater, gently dried and fresh frozen for shipment to the lab. In this farm, 50 broodstock were also analysed. These animals neither showed symptoms of LCD nor had LCD history, as stated by the farm records. For sampling purposes, these specimens were anaesthetized with MS-222 in seawater at a final concentration of 30 mg/ml. For each specimen, samples of caudal fin and blood were obtained. Blood samples were collected from the branchial arches using S-Monovette 4.5 ml LH (Sarstedt, Nümbrecht, Germany), chilled to 4 °C and sent to the lab for analysis within 24 h, whereas samples of caudal fin were obtained and stored as described above.

Samples were homogenized in Leibovitz’s L-15 medium (Gibco, Life Technologies, Carlsbad, CA, USA) (10 % w/v) as described previously [27], except samples of eggs, rotifers and artemia, which were ground in liquid nitrogen. Total DNA was extracted from 200 μl of tissue homogenates or 50 mg of tissue powder using the QIAamp DNA Minikit (Qiagen, Valencia, CA, USA) according to the manufacturer’s instructions. Finally, DNA was extracted from 200 μl of heparinised blood using the ReliaPrep Blood gDNA Miniprep System (Promega, Madison, WI, USA). In all cases, DNA was eluted in a final volume of 100 μl and stored at -20 °C until use as template for PCR-hybridization and qPCR. Prior to PCR assays, purified DNA was quantified spectrophotometrically using a NanoDrop 1000 spectrophotometer (Thermo Scientific, West Palm Beach, FL, USA), and DNA was diluted to achieve a final concentration of 20 ng/μl.

### PCR-hybridization

LCDV genome was detected using the PCR-hybridization assay described by Cano et al. [19]. Briefly, a 270-bp fragment of the MCP gene of LCDV was amplified by PCR. PCR products were denatured and blotted onto a Hybond-N nylon membrane (GE Healthcare, Waukesha, WI, USA), and hybridization was carried out using an LCDV-specific DIG-labelled probe and the chemiluminescent substrate CSPD (Roche Applied Science, Mannhein, Germany).

### Cloning a fragment of MCP gene

Lymphocystis disease virus isolate SA9 was used as source of LCDV DNA [14]. A fragment of the viral MCP gene was amplified by PCR using the primers LCDVs-F and LCDVs-R described by Kitamura et al. [12]. PCR was performed in a 50-μl reaction volume containing 10 μl of 5X Colorless GoTaq Flexi Buffer (Promega), 3 mM MgCl_2_ (Promega), 5 μl of 0.2 mM dNTP (Roche), 1.25 U of GoTaq DNA polymerase (Promega), and 2 μl of each primer at 15 pmol/μl. DNA was amplified by the use of one denaturation step at 95 °C for 5 min, followed by 35 cycles of denaturation (95 °C for 1 min), annealing (50 °C for 30 s), and extension (72 °C for 1 min), and a final extension step at 72 °C for 10 min. PCR products were run in 2 % agarose gels, purified using the HighPure PCR Product Purification kit (Roche), and cloned into the pCR4-TOPO vector (TOPO TA cloning kit) (Invitrogen, Life Technologies, Carlsbad, CA, USA) for subsequent transformation in *Escherichia coli* (One Shot competent cells, Invitrogen) following manufacturer’s instructions. Recombinant plasmid DNA was purified from *E. coli* cells with a commercial kit (High Pure Plasmid Isolation kit, Roche), and insert size was verified by PCR and sequencing, using the M13 primers provided in the cloning kit. The cloned MCP gene fragment was 609 bp in length, corresponding with nucleotide positions 99 to 707 of the LCDV SA9 MCP gene (GenBank accession no. GU320728).

### Real-time PCR assay

Primers for qPCR (RT-LCDV-F: 5′-ACGTTTCTCGAGGCGGAGAT-3′, and RT-LCDV-R: 5′-CGGACGTTTGCTTGACCAA -3′) were designed to target the MCP gene of LCDV genotype VII, using Primer Express Software v3.0 (Applied Biosystems, Life Technologies, Carlsbad, CA, USA). This primer set generates a 150-bp amplicon within the 609-bp cloned fragment of MCP gene (nucleotide positions 173 to 322 of the LCDV SA9 MCP gene).

Real-time PCR reactions were carried out in 96-well plates (Applied Biosystems), in a final volume of 50 μl containing 25 μl of 2x Power SYBR Green PCR Master Mix (Applied Biosystems), 3 μl of each primer at 15 pmol/μl, and 10 μl of DNA. PCR amplifications were performed in a 7500 Real-Time PCR System (Applied Biosystems). The thermal profile was: 50 °C for 2 min; 95 °C for 10 min, and 40 cycles at 95 °C for 15 s and 60 °C for 1 min. Finally, dissociation curve analysis was carried out automatically in order to allow detection of non-specific amplification products.

### Standard curve for LCDV quantification

To quantify the amount of viral DNA in different samples, a standard curve was generated using the recombinant plasmid described above. The concentration of the purified plasmid was determined by spectrophotometry as described above, and the plasmid stock was diluted to serve as template for qPCR (10-fold serial dilutions ranging from 10^6^ to 10^2^ copies, and then two-fold dilutions from 50 to 25 copies, and from 16 copies to 1 copy).

The sensitivity of the qPCR assay was also determined in terms of infective viral particles. LCDV SA9 stock was titrated in SAF-1 cells, using the 50 % cell culture infectious dose (TCID_50_) endpoint dilution assay as described previously [27]. An aliquot of 1 ml of the viral stock with a titre adjusted to 1 x 10^6^ TCID_50_/ml was subjected to a 10-fold serial dilution in Leibovitz’s L-15 medium. The DNA of each dilution was extracted as previously specified, and used for qPCR. The amount of infective virus analysed per reaction was 1 x 10^5^ to 1 x 10^0^ TCID_50_.

Milli-Q water and DNA from one LCDV-negative gilthead seabream sample [19] were included within each qPCR run as no-template and negative controls, respectively. In each 96-well, plasmid dilutions for the standard curve were run along with the samples and controls, using three technical replicates. The number of copies of LCDV DNA in each well was calculated from its cycle threshold (C_t_) value by interpolation in the standard curve (C_t_*versus* log copy number), using the SDS Software v1.3 (Applied Biosystem). The amplification efficiency (*E*) was calculated from standard curves using the formula *E* = (10^-1/S^ - 1) x 100 (*S* being the slope of the linear fit). Viral loads in samples were calculated as the mean of the three replicates, and expressed as viral DNA copies per milligram of tissue (per μl in blood samples).

### Assay repeatability and reproducibility

To evaluate the precision of the qPCR, the intra- and inter-assay variability was determined using the recombinant plasmid. To assess intra-assay variation, four plasmid DNA dilution series (from 10^5^ to 2 copies per reaction) were prepared and tested simultaneously in the same plate. Four separate PCR runs were carried out to assess inter-assay variation, using also seven dilutions of plasmid DNA. The mean, standard deviation (SD) and coefficient of variation (CV) were calculated independently for each DNA dilution.

## Results

### Evaluation of the real-time PCR assay

Specificity of the qPCR was determined by analysis of the dissociation curves generated in each experiment. Standard and positive samples gave a single PCR product with a melting temperature of 77.7 ± 0.5 °C, which correspond with that deduced from the sequence of the expected fragment. The size of amplicons was monitored by agarose gel electrophoresis, and bands were observed at the expected size (150 bp).

**Fig. 1:**
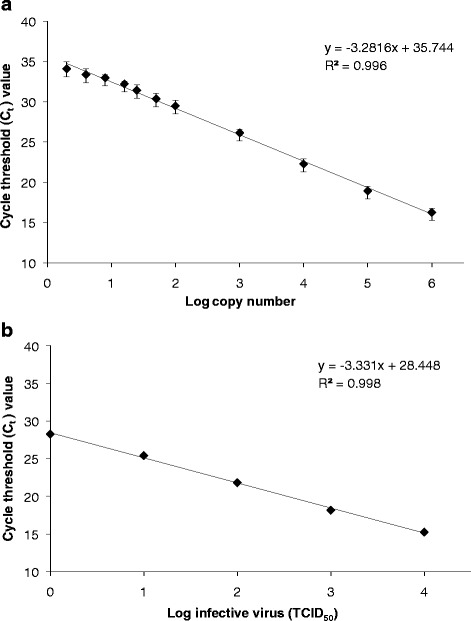
Dynamic range and sensitivity of the real-time PCR assay for LCDV detection. **a** Standard curve obtained using dilutions of plasmid DNA ranging from 10^6^ to 2 copies per reaction. Linear regression was performed on mean data from four separate runs. The logarithm to base 10 (log) of the plasmid copy number *versus* the cycle threshold (C_t_) value is represented. **b** Standard curve showing a linear relationship between the log of the amount of infective virus (expressed in TCID_50_) per reaction and their corresponding C_t_ values

The linear dynamic range, efficiency and precision of the qPCR assay were evaluated using a recombinant plasmid containing a 609-bp fragment of the LCDV MCP gene. Standard curves generated from four independent assays demonstrated a linear relationship between the amount of plasmid DNA and C_t_ values over a wide range of concentration, from 10^6^ copies (C_t_ = 16.25 ± 0.48) to 2 copies (C_t_ = 34.09 ± 1.14) per reaction (Fig. 1a). The regression analysis yielded a correlation coefficient (*r*) ≥ 0.994 and an amplification efficiency of 101.89 ± 5.11 %. The mean intra-assay variation was 1.38 ± 0.87 % when analysing four replicates of plasmid dilutions, whilst the mean inter-assay variation among four experiments was 2.63 ± 0.48 % (Table 1). These CV values were considered acceptable to validate the repeatability and reproducibility of the assay.

**Table 1:** Intra- and inter-assay variability of the real-time PCR

	Intra-assay variation^a^		Inter-assay variation^b^	
Copies/reaction	Cycle threshold (C_t_)^c^	CV (%)	Cycle threshold (C_t_)^c^	CV (%)
1 x 10^5^	19.92 ± 0.16	0.78	18.92 ± 0.54	2.83
1 x 10^4^	23.34 ± 0.17	0.73	22.27 ± 0.60	2.69
1 x 10^3^	26.87 ± 0.18	0.66	26.14 ± 0.46	1.75
1 x 10^2^	30.22 ± 0.27	0.89	29.49 ± 0.71	2.39
1.6 x 10^1^	32.15 ± 0.47	1.45	32.22 ± 0.87	2.72
4 x 10^0^	34.15 ± 0.80	2.34	33.37 ± 0.89	2.68
2 x 10^0^	35.23 ± 0.99	2.80	34.09 ± 1.14	3.35
Overall CV^c^ (%)		1.38 ± 0.87		2.63 ± 0.48

^a^ Intra-assay variation was determined on four replicates of recombinant plasmid dilutions analysed in the same PCR run

^b^ Inter-assay variation was calculated on values obtained in four separate PCR runs

^c^ Mean ± standard deviation (SD)

*CV* coefficient of variation

A linear relationship between the infective titre of a viral suspension and C_t_ values was also observed for viral amounts ranging from 1 x 10^4^ to 1 x 10^0^ TCID_50_ per reaction (*r* = 0.999) (Fig. 1b), with 1 x 10^0^ TCID_50_ yielding a mean viral DNA copy number of 1.2 x 10^2^.

### Surveillance study of LCDV in gilthead seabream farms

In the present study, individual gilthead seabreams were collected from four farms with different background regarding to LCD. Farm C had no records of LCD, either before or after this study, whereas farm D suffered LCD outbreaks in last years. In farms A and B an outbreak of LCD was going on at the time of sampling, affecting several tanks. In these latter farms, fish from two different cycles of production were sampled, one suffering and other unaffected by LCD.

A total of 60 juvenile fish, both asymptomatic and diseased, were analysed by the qPCR assay developed in this study. LCDV was detected in all farms, 30 to 100 % of fish being identified as LCDV-infected (Table 2). In parallel, asymptomatic fish were also analysed by PCR-hybridization, corroborating in all cases their infection status. In asymptomatic fish, viral loads in caudal fin ranged between 1 copy and 3.3 x 10^2^ copies of viral DNA per mg of tissue, whereas in animals with signs of the disease, viral loads were between 2.9 x 10^4^ and 6.6 x 10^5^ copies of viral DNA/mg.

**Table 2:** LCDV detection and estimated viral load determined by real-time PCR in caudal fin from diseased and asymptomatic gilthead seabream juveniles

			Viral load^d^	
Fish population^a^	LCD signs^b^	LCDV detection^c^	Load range	Mean ± SD
A	-	73.3 (15)	1–15	(4 ± 3.99) x 10^0^
A	+	100 (6)	1.2 x 10^5^–6.6 x 10^5^	(3.53 ± 2.77) x 10^5^
B	-	30.0 (10)	1–330	(1.1 ± 1.89) x 10^2^
B	+	100 (5)	2.9 x 10^4^–6.9 x 10^4^	(5 ± 1.75) x 10^4^
C	-	87.5 (16)	1.7–54	(1.6 ± 1.56) x 10^1^
D	-	100 (8)	12–140	(5.3 ± 5.88) x 10^1^

^a^ A, B, C and D are four gilthead seabream farms located in the Mediterranean area. Only farm C had no previous reports of lymphocystis disease (LCD)

^b^ Typical lymphocystis disease signs: +, presence; -, absence

^c^ Percentage of LCDV-positive fish (total number of fish analysed)

^d^ Copies of viral DNA per mg of tissue

*SD* standard deviation

Samples were also collected at the hatchery in another farm with previous LCD records (farm E). Rotifer and artemia metanauplius cultures, and artemia cysts were positive for LCDV. Viral loads estimated for these samples were about 10^2^ copies of viral DNA per mg (Table 3). In addition, one sample of decapsulated artemia cysts was analysed, and found to be LCDV-positive (1.2 x 10^1^ copies of viral DNA/mg). LCDV was also detected by qPCR in non-disinfected gilthead seabream fertilized eggs, in animals collected at different tanks in the larval rearing unit (1- to 26-d-old larvae), and in fingerlings collected in the weaning area. Viral loads in these gilthead seabream samples ranged from 1.3 x 10^0^ to 2.2 x 10^1^ copies of viral DNA/mg. All the samples collected at this hatchery were also found to be LCDV-positive by PCR-hybridization.

**Table 3:** Estimated viral load determined by real-time PCR in samples from a gilthead seabream hatchery

Sample^a^	Viral load^b^
Rotifer culture	1.5 x 10^2^
Artemia cysts	1.9 x 10^2^
Decapsulated artemia cysts	1.2 x 10^1^
Artemia metanauplii	1.5 x 10^2^
Fertilized eggs	4.1 x 10^0^
Larvae (1-d-old)	3.4 x 10^0^
Larvae (6-d-old)	2.5 x 10^0^
Larvae (8-d-old)	3.6 x 10^0^
Larvae (26-d-old)	5.1 x 10^0^
Fingerlings (0.3 g)	1.1 x 10^1^
Fingerlings (0.9 g)	1.3 x 10^0^
Fingerlings (1.6 g)	2.2 x 10^1^
Fingerlings (2 g)	9.7 x 10^0^

^a^ Samples consisted of pooled animals

^b^ Copies of viral DNA per mg of tissue

Finally, broodstock from farm E were analysed using the qPCR assay (Table 4). LCDV was detected in 22 out of the 50 caudal fin samples analysed, with estimated viral loads ranging from 8.7 to 61 copies of viral DNA/mg. When blood samples were considered, only 9 of the fish were identified as LCDV-positive (1.3 ± 1.37 copies of viral DNA/μl).

**Table 4:** LCDV detection and estimated viral load determined by real-time PCR in caudal fin and blood from asymptomatic gilthead seabream breeders

		Viral load^b^	
Samples	LCDV detection^a^	Load range	Mean ± SD
Caudal fin	44 (50)	8.7–61	(2.7 ± 1.40) x 10^1^
Blood	18 (50)	0.2–4.4	(1.3 ± 1.37) x 10^0^

^a^ Percentage of LCDV-positive fish (total number of fish analysed)

^b^ Copies of viral DNA per mg of tissue (caudal fin samples) or per μl (blood samples)

*SD* standard deviation

## Discussion

LCD outbreaks are frequently observed in the Mediterranean gilthead seabream aquaculture. Although it is usually described as a self-limiting disease, there are several reports on mortalities up to 45 % in juvenile fish, which may be related to secondary bacterial infections or with particularly large growth of lymphocysts, which severely impaired fish breathing or feeding [4, 10, 28]. As no effective treatments or commercially available vaccines currently exist, LCD prevention in hatcheries must rely on the selection of LCDV-free broodstock, the use of effective decontamination methods to prevent viral transmission from asymptomatic broodstock to larvae, and the supply of virus - freelive food [17, 22, 29]. During the growing period, the selection of non-infected fish is also advisable, as LCDV-positive juveniles may become symptomatic under stress conditions such us transport to on-growing facilities. Moreover, little information is available on a number of epidemiological questions concerning LCDV infections, as the number of virus particles required to induce the disease, the number of genome copies present in asymptomatic and diseased fish, and the kinetics of viral replication.

Previous studies have demonstrated the applicability of a PCR-based method to detect LCDV in asymptomatic gilthead seabream carriers, independently of its size, as well as in rotifer and artemia cultures [17, 19, 22]. Nevertheless, this method is relatively time-consuming, as an additional step of blot-hybridization of the PCR products is required to detect LCDV-positive samples. Furthermore, this assay is not quantitative and applicable for routine diagnosis.

In the present study a qPCR assay has been developed and applied to detect and quantify LCDV in different samples. The assay was specific for LCDV, as demonstrated by analysis of the melting curves generated from each sample. Its analytical sensitivity, determined as the smallest copy number of the plasmid standard reliably detected, was 2 copies of DNA per reaction. The qPCR assay also showed a wide linear dynamic range, extending to 6 log_10_ concentrations of plasmid DNA, and infective titres from 10^4^ to 1 TCID_50_. In addition, the precision of the assay was supported by the high correlation coefficients obtained for the standard curves, and the intra- and inter-assay variation of C_t_ values.

Using the protocol as described, the qPCR assay allows the detection of the virus at levels as low as 1 copy of viral DNA per mg of fish tissue. This high sensitivity, combined with its wide dynamic range, makes the qPCR assay suitable to detect low viral loads in subclinical LCDV infections, and, at the same time, to quantify variable viral loads in the course of infection. In addition, it could be useful in searching for potential LCDV reservoirs.

The application of the qPCR assay to LCDV surveillance in fish farms has shown that monitoring the infection in individual fish, both diseased and subclinically infected, is possible by sampling caudal fin as reported previously [16, 19]. The prevalence of LCDV infection in the asymptomatic gilthead seabream populations analysed varied from 30 to 100 %, even in one farm with no previous LCD records. In these fish, estimated viral load in caudal fin was two to five orders of magnitude lower than in diseased fish. Thus viral load seems to correlate with disease manifestation. However, if the low viral loads detected in subclinical infections represent a status associated with viral replication remains to be determined. In addition, the qPCR assay developed could be a valuable tool to study the correlation between viral multiplication and the onset of symptoms in experimental LCDV infections.

Palmer et al. [15] developed a real-time PCR method using fluorogenic primers, specific for LCDV genotype IX sequences, which proved to be reliable in the detection of subclinically infected yellow perch, although its sensitivity was 5 x 10^2^ copies of DNA per mgL^-1^. LCDV quantification by qPCR has also been carried out in a reduced number of samples from diseased and recovered gilthead seabream [26]. The analytical sensitivity of the former assay was 5.2 copies of DNA per reaction that is more than twice the value reported in the present study. Furthermore, although the authors reported some quantitative data, viral loads were not expressed in terms of viral DNA copies per amount of tissue, which prevents further comparisons [15, 26].

Carrier fish were also identified in the broodstock from a farm with LCD records by analysing caudal fin samples by qPCR. The assay was applied in parallel to blood samples, and although LCDV could be detected, estimated viral load, and also clinical sensitivity, was lower than that obtained in caudal fin analysis. In this farm, the q-PCR assay allowed the quantitative detection of LCDV in all samples collected in the hatchery, including fertilized eggs, larvae and fingerlings, and also rotifer cultures and artemia metanauplii and cysts used for larval rearing. In these samples, as well as in caudal fin samples from asymptomatic juvenile fish, the qPCR assay showed the same clinical sensitivity than the PCR-hybridization protocol described by Cano et al. [19], but is completed in 130 min, including melting curve generation, which considerably reduces the time required for LCDV diagnosis. In addition, the results of this study support the existence of multiple reservoirs of LCDV in the farm facilities, as previously suggested by Cano et al. [17, 22], and the importance of proper application of effective disinfection treatments, as those recommended by the FAO [30], to avoid viral transmission through fish eggs or live food.

## Conclusions

The qPCR assay developed in this study is a sensitive, specific and reliable method for detection and quantification of LCDV in gilthead seabream. The method is rapid and appropriate for LCDV surveys for which the detection of subclinical LCDV infections in carrier fish is essential. The assay could also be valuable to identify LCDV reservoirs or to study viral replication in fish.

### Availability of data

All the data supporting the results are included in the article.

## References

[1] Anders K, Ahne W, Kurstak D (1989). Lymphocystis disease of fishes. Viruses of lower vertebrates.

[2] Menezes J, Ramos MA, Pereira TG (1987). Lymphocystis disease: an outbreak in *Sparus aurata* from Ria Formosa, south coast of Portugal. Aquaculture.

[3] Borrego JJ, Castro D, Balebona MC, Garcia-Rosado E, Lopez-Cortes L (2001). Patologías que afectan al cultivo de la dorada (*Sparus aurata*, L.) en la Comunidad Autónoma andaluza.

[4] Colorni A, Padrós F, Pavlidis M, Mylonas C (2011). Diseases and health management. Sparidae: biology and aquaculture of gilthead sea bream and other species.

[5] Wolf K (1988). Fish viruses and fish viral diseases.

[6] Smail DA, Munro ALS, Roberts RJ (2001). The virology of teleosts. Fish pathology.

[7] Masoero L, Ercolini C, Caggiano M, Rossa A (1986). Osservazioni preliminari sulla linfocisti in una maricoltura intensive italiana. Riv Ital Piscicul Ittiopatol.

[8] Basurco A, Marcotegui MA, Rueda A, Tidana A, Castellanos A, Tarazona JV (1990). First report of lymphocystis disease in *Sparus aurata* (Linnaeus) in Spain. Bull Eur Ass Fish Pathol.

[9] Moate RM, Harris JE, McMahon S (1992). Lymphocystis infections in cultured gilt-head sea bream (*Sparus aurata*) in the Aegean sea. Bull Eur Ass Fish Pathol.

[10] Haddad-Boubaker S, Bouzgarou N, Fakhfakh E, Khayech M, Mohamed SB, Megdich A, Chéhida NB (2013). Detection and genetic characterization of lymphocystis disease virus (LCDV) isolated during disease outbreaks in cultured gilt-head sea bream *Sparus aurata* in Tunisia. Fish Pathol.

[11] Kitamura SI, Jung SJ, Kim WS, Nishizawa T, Yoshimizu M, Oh MJ (2006). A new genotype of lymphocystivirus, LCDV-RF, from lymphocystis diseased rockfish. Arch Virol.

[12] Kitamura SI, Jung SJ, Oh MJ (2006). Differentiation of Lymphocystis disease virus genotype by multiplex PCR. J Microbiol.

[13] Hossain M, Song JY, Kitamura SI, Jung SJ, Oh MJ (2008). Phylogenetic analysis of lymphocystis disease virus from tropical ornamental fish species based on a major capsid protein gene. J Fish Dis.

[14] Cano I, Valverde EJ, Lopez-Jimena B, Alonso MC, Garcia-Rosado E, Sarasquete C (2010). A new genotype of *Lymphocystivirus* isolated from cultured gilthead seabream, *Sparus aurata* L., and Senegalese sole, *Solea senegalensis* (Kaup). J Fish Dis.

[15] Palmer LJ, Hogan NS, van den Heuvel MR (2012). Phylogenetic analysis and molecular methods for the detection of lymphocystis disease virus from the yellow perch, *Perca flavescens* (Mitchell). J Fish Dis.

[16] Kvitt H, Heinisch G, Diamant A (2008). Detection and phylogeny of *Lymphocystivirus* in sea bream *Sparus aurata* based on the DNA polymerase gene and major capsid protein sequences. Aquaculture.

[17] Cano I, Valverde EJ, Garcia-Rosado E, Alonso MC, Lopez-Jimena B, Ortiz-Delgado JB (2013). Transmission of lymphocystis disease virus to cultured gilthead seabream, *Sparus aurata* L., larvae. J Fish Dis.

[18] Sanz F, Coll J (1992). Techniques for diagnosing viral diseases of salmonid fish. Dis Aquat Org.

[19] Cano I, Ferro P, Alonso MC, Bergmann SM, Römer-Oberdörfer A, Garcia-Rosado E (2007). Development of molecular techniques for detection of lymphocystis disease virus in different marine fish species. J App Microbiol.

[20] Zan J, Sun X, Zhang Z, Qu L, Zhang J (2007). Application of quantitative PCR method in detection of lymphocystis disease virus China (LCDV-cn) in Japanese flounder *(Paralichthys olivaceus*). Chin J Oceanol Limnol.

[21] Hossain M, Kim SR, Kitamura SI, Kim DW, Jung SJ, Nishizawa T (2009). Lymphocystis disease virus persists in the epidermal tissues of olive flounder, *Paralichthys olivaceus* (Temminch & Schlegel), at low temperatures. J Fish Dis.

[22] Cano I, Lopez-Jimena B, Garcia-Rosado E, Ortiz-Delgado JB, Alonso MC, Borrego JJ (2009). Detection and persistence of Lymphocystis disease virus (LCDV) in *Artemia* sp. Aquaculture.

[23] Pallister J, Gould A, Harrison D, Hyatt A, Jancovich J, Heine H (2007). Development of real-time PCR assays for the detection and differentiation of Australian and European ranaviruses. J Fish Dis.

[24] Pepin JF, Riou A, Renault T (2008). Rapid and sensitive detection of ostreid herpesvirus 1 in oyster samples by real-time PCR. J Virol Methods.

[25] Cutrín JJ, Olveira JG, Bandín I, Dopazo CP (2009). Validation of a real time RT-PCR applied to cell culture for diagnosis of any known genotype of viral haemorrhagic septicaemia virus. J Virol Methods.

[26] Ciulli S, Pinheiro AC, Volpe E, Moscato M, Jung TS, Galeotti M (2015). Development and application of a real-time PCR assay for the detection and quantitation of lymphocystis disease virus. J Virol Methods.

[27] Alonso MC, Cano I, Garcia-Rosado E, Castro D, Lamas J, Barja JL, Borrego JJ (2005). Isolation of lymphocystis disease virus from sole, *Solea senegalensis* Kaup, and blackspot sea bream, *Pagellus bogaraveo* (Brünnich). J Fish Dis.

[28] Dezfuli BS, Lui A, Giari L, Castaldelli G, Mulero V, Noga EJ (2012). Infiltration and activation of acidophilic granulocytes in skin lesions of gilthead seabream, *Sparus aurata*, naturally infected with lymphocystis disease virus. Dev Comp Immunol.

[29] Yoshimizu M (2009). Control strategy for viral diseases of salmonid fish, flounders and shrimp at hatchery and seed production facility in Japan. Fish Pathol.

[30] Moretti A, Fernandez-Criado MP, Cittolin G, Guidastri R (1999). Manual on hatchery production of seabass and gilthead seabream.

